# Progressive Supranuclear palsy (PSP) disease progression, management, and healthcare resource utilization: a retrospective observational study in the US and Canada

**DOI:** 10.1186/s13023-024-03168-z

**Published:** 2024-05-23

**Authors:** Ella Nysetvold, Lauren N. Lopez, Ashley N. Cogell, Henrik Fryk, Nelson D. Pace, Sara Snell Taylor, Joyce Rhoden, Caitlin A. Nichols, Demetris Pillas, Alexander Klein, Teresa Gasalla, Anna Scowcroft

**Affiliations:** 1 AllStripes Research, San Francisco, California USA; 2 UCB Pharma, Brussels, Belgium; 3 Gillings School of Global Public Health, University of North Carolina at Chapel Hill, Chapel Hill, North Carolina, USA

**Keywords:** Progressive supranuclear palsy, PSP, Real-world data, Patient recruitment, Healthcare resource utilization, Observational study, Patient registry, Patient-centric, Epidemiology

## Abstract

**Background:**

Progressive supranuclear palsy (PSP) is a rare neurodegenerative brain disease with rapid progression and currently limited treatment options. A comprehensive understanding of disease progression, management, and healthcare resource utilization is limited, and further research is challenging due to the small population of patients. To address these challenges in conducting PSP research, individuals with PSP were recruited using a multichannel approach tailored specifically to the PSP community. We performed a retrospective observational study using data abstracted from participant medical records collected from multiple patient care centers.

**Results:**

Seventy-two individuals with PSP were eligible for inclusion. On average, 144 medical documents per participant were collected from an average of 2.9 healthcare centers per participant, with a mean study period of 7.9 years. Among participants with a date of symptom onset documented in the medical records, the median time for the onset of the first fall was 2.0 years (IQR 3.2) before diagnosis, the median onset of unsteady gait or gait impairment was 1.2 years (IQR 1.8) before diagnosis, and the median onset of mobility problems was 0.8 years (IQR 1.8) before diagnosis. The most widely utilized healthcare resources, with at least 85% of participants using each of these resources at some point during the disease course, were medications (100%), imaging (99%), assistive devices (90%), supportive care (86%), and surgeries and procedures (85%).

**Conclusions:**

This retrospective study adds to the current understanding of PSP symptoms, comorbidities, and healthcare resource utilization (HRU) across the disease journey. By involving individuals with PSP and their caregivers or legally authorized representatives in the research process, this study was unique in its approach to participant recruitment and enabled individuals to participate in research without the need for travel. We collected medical documents from multiple healthcare centers, allowing for broad data collection covering the entire disease journey. This approach to the collection of real-world data may be used to generate valuable insights into many aspects of disease progression and management in PSP and many other rare diseases.

**Supplementary Information:**

The online version contains supplementary material available at 10.1186/s13023-024-03168-z.

## Background

Progressive supranuclear palsy (PSP) is a rare, progressive, and ultimately fatal neurodegenerative brain disease. PSP symptoms at presentation are heterogeneous, and early symptoms may include balance-related issues and falling, problems with vision, akinesia, and cognitive dysfunction [1, 2]. Despite advancements in understanding the disease [3], treatment options are limited to symptomatic and supportive therapies, and there remains no approved disease-modifying treatment for PSP [2]. Life expectancy after disease onset is approximately 7–8 years [1, 4], yet individuals often do not receive a PSP diagnosis for several years after the onset of symptoms [2, 4].

In addition to the need for accurate and timely diagnosis of PSP, understanding disease progression is a concern for patients, caregivers, and physicians, especially given the lack of disease-modifying treatments. Greater understanding of disease progression and management under the current framework of care can provide insight into the burden of illness and highlight any limitations and unmet patient needs under current care practices. This understanding is also important for clinical trial design and treatment development and may further facilitate discussions of advanced care planning at earlier stages of disease.

Due to the small number and wide geographical distribution of rare disease patients, recruitment for research studies is challenging, and traditional recruitment methods often have limited success [5–7]. Recruitment for research studies in PSP presents additional challenges due to the older age at PSP diagnosis and the rapid progression of the disease. Consequently, traditional prospective natural history studies for PSP with site-based recruitment are often limited by low participant numbers [1, 8, 9], while larger studies [10, 11] are often expensive and time-consuming to run, and participation can be burdensome for patients [7].

Real-world data (RWD), which are collected outside of a clinical trial [12], such as claims databases, electronic health records (EHRs), or medical records, present an alternative approach to conducting research into rare diseases. Several studies of PSP symptoms and progression have been conducted using claims [13–15] and EHR data [16, 17]; however, these studies are impacted by the inherent limitations of these data sources. Claims data only include insured patients and are dependent on the accurate use of billing codes for visits and procedures. EHR data are limited to only specific healthcare centers using a given EHR system and therefore may not capture the full patient journey across all healthcare settings when a patient receives care at centers using different EHR systems. Furthermore, both claims and structured EHR data lack the clinical depth needed to fully understand the nuances of treatment decisions that impact a patient’s healthcare journey and disease progression.

Patient medical records are an alternative source of RWD that provides both structured data on test results, medications, and healthcare utilization, as well as unstructured narrative text from physician notes on treatment decisions, clinical progress notes, and other results that are lacking from claims and EHR data. Medical records can be collected from every care setting ranging from large academic institutions to local community care centers, ensuring data capture across the spectrum of settings in which patients receive health care. To date, however, few studies have leveraged data abstracted directly from full medical records, including structured and unstructured data, as an RWD source for research to improve the understanding of PSP progression and management [4, 18–21]. The studies that have used these data often rely on a small number of sites for recruitment and record collection, limiting the breadth of data to clinical encounters at larger medical centers and thereby excluding care received in the community or at nonparticipating specialist centers from the patient journey.

To bridge the current gap in understanding PSP progression and management across the full patient journey and spectrum of healthcare settings, this study leveraged data from medical records collected from multiple healthcare centers at which individuals with PSP or their legally authorized representatives reported receiving care. The key aims of this study were 1) to use novel methods of patient recruitment and engagement to establish a recontactable cohort of research participants with PSP from across the US and Canada and 2) to conduct a retrospective observational study within this cohort to build upon the literature on PSP disease characteristics, including symptoms, comorbidities, and healthcare resource utilization (HRU).

## Results

### Participant recruitment

Our program recruited 122 individuals with PSP within 21 months, of whom 72 participants from the US and Canada were eligible for inclusion in this study. Of these, 20 individuals chose to become AllStripes Ambassadors to raise awareness of the research program and encourage other individuals in their communities to join the research program.

### Record collection and demographics

**Fig. 1:**
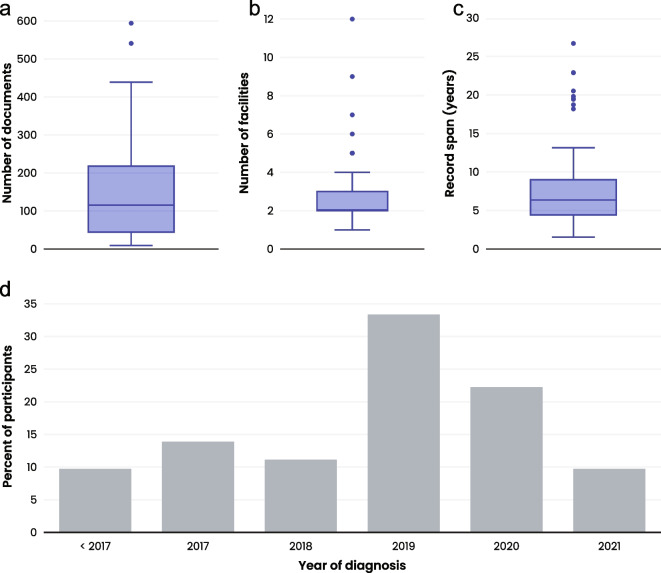
Records Collection. Box plots presenting (**a**) Number of medical documents per participant (*n* = 72); **b** Number of healthcare facilities per participant (*n* = 72); **c** Duration of record span (years) per participant (*n* = 72). Box and whisker plots (**a-c**) show 50% of the data within the box. The bottom of the box represents the 25th percentile (Q1), the top of the box represents the 75th percentile (Q3), and the solid line inside the box represents the median (Q2). The whiskers below the box indicate Q1–1.5*IQR, and the whiskers above the box represent Q3 + 1.5*IQR. Data points outside the box and whisker represent outliers. **d** Bar graph presenting the year of diagnosis (*n* = 72)

A cohort of 72 participants from the AllStripes program were screened in this study based on the inclusion criteria described in the Methods section below. In total, > 10,000 medical documents were collected for these 72 participants from 194 unique healthcare centers in the US and Canada, with a mean of 144.4 documents (median: 115.5, IQR: 172.2) per participant from 2.9 healthcare centers (median: 2.0, IQR: 1.0) per patient (Fig. 1A, B). The mean length of follow-up per participant from the earliest to most recent record was 7.9 years (median: 6.4 years, IQR: 4.5 years), with a mean of 1.9 years of follow-up after diagnosis (median: 1.5 years, IQR: 1.7 years) (Fig. 1C). Five participants had less than 1 year of records prior to diagnosis, and 21 participants had less than 1 year of records after diagnosis. Participant PSP diagnosis dates ranged from 2011 to 2021. The majority of participants were female (*n* = 41, 56.9%) and US-based (*n* = 62, 86.1%), with a median age at the end of the study period of 71.3 years (IQR: 11.9 years) and a median age at diagnosis of 69.3 years (IQR: 11.5 years) (Table 1). Most individuals did not have clinical documentation of a predominant PSP phenotype available in their medical records (*n* = 60, 83.3%), and of those who did have a documented phenotype, PSP-RS (PSP-Richardson’s syndrome) was the most common (*n* = 7/12, 53.8%). At the end of the study period, three participants were deceased; 18 were in their first year following diagnosis; 26 were between one and 2 years postdiagnosis; and 25 were more than 2 years postdiagnosis (Table 1).

**Table 1:** Participant demographic characteristics (*n* = 72)

Characteristic		*N* = 72
**Sex**		
	Female	41 (57%)
	Male	31 (43%)
**Country**		
	Canada	10 (14%)
	United States	62 (86%)
**Median age at end of study period**		71.3 [51.3, 83.0]
**Age at end of study period**		
	< 60	6 (8%)
	60–69	23 (32%)
	70–79	40 (56%)
	80–89	3 (4%)
**Median age at diagnosis**		69.3 [49.6, 82.0]
**Age at diagnosis**		
	< 60	13 (18%)
	60–69	25 (35%)
	70–79	32 (44%)
	80–89	2 (3%)
**Status at end of study period**		
	Less than 1 year postdiagnosis	18 (25%)
	1 year postdiagnosis	26 (36%)
	2 years postdiagnosis	10 (14%)
	3 years postdiagnosis	11 (15%)
	More than 3 years postdiagnosis	4 (6%)
	Deceased	3 (4%)

n (%); median [range]

Sex, country, and age were self-reported or reported by the participant’s legally authorized representative at study enrollment. When characteristics were missing or unknown from incomplete enrollment surveys, medical records were used as a secondary source for completing the information shown in the table. Age at diagnosis, age at end of study period, and participant status at end of study period were derived from medical record data

### PSP symptoms and comorbidities

**Fig. 2:**
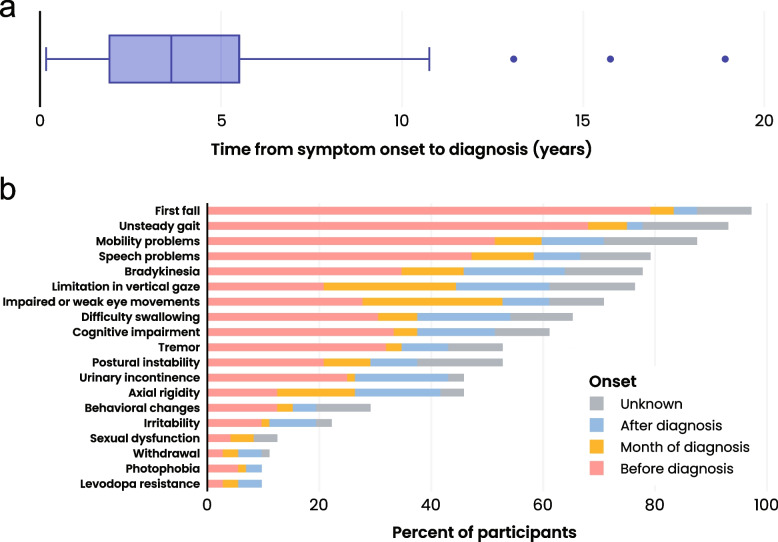
PSP Symptoms. **a** Box plot presenting time from first symptom onset to diagnosis (*n* = 70). The date of symptom onset was unknown for two participants. The box plot shows 50% of the data within the box. The bottom of the box represents the 25th percentile (Q1), the top of the box represents the 75th percentile (Q3), and the solid line inside the box represents the median (Q2). The whiskers below the box indicate Q1–1.5*IQR, and the whiskers above the box represent Q3 + 1.5*IQR. Data points outside the box and whisker represent outliers. **b** Bar chart presenting common symptoms of PSP by onset before diagnosis (pink), month of diagnosis (yellow), after diagnosis (blue), or unknown onset (gray) (*n* = 72)

Almost all (97.2%) participants had at least one symptom documented in the medical record prior to diagnosis, with a mean time from recorded symptom onset to diagnosis of approximately 4.2 years (median: 3.7 years, IQR: 3.5) (Fig. 2A). Falling (*n* = 57, 79.2%), unsteady gait or gait impairment (*n* = 49, 68.1%), and mobility problems (*n* = 37, 51.4%) were the most common symptoms presenting during the prediagnosis period (Fig. 2B, Additional file 2).

Nearly all study participants experienced the symptom of falling (*n* = 70, 97.2%) at some point during their disease course. More than half of the study participants had documentation of unsteady gait/gait impairment (*n* = 67, 93.1%), mobility problems (*n* = 63, 87.5%), speech problems/slurred speech/dysarthria (*n* = 57, 79.2%), bradykinesia (*n* = 56, 77.8%), limitation in vertical gaze (*n* = 55, 76.4%), impaired or weak eye movements/saccades (*n* = 51, 70.8%), difficulty swallowing/dysphagia (*n* = 47, 65.3%), cognitive impairment (*n* = 44, 61.1%), postural instability (*n* = 38, 52.8%), and/or tremor (*n* = 38, 52.8%) (Fig. 2B, Additional file 2).

**Fig. 3:**
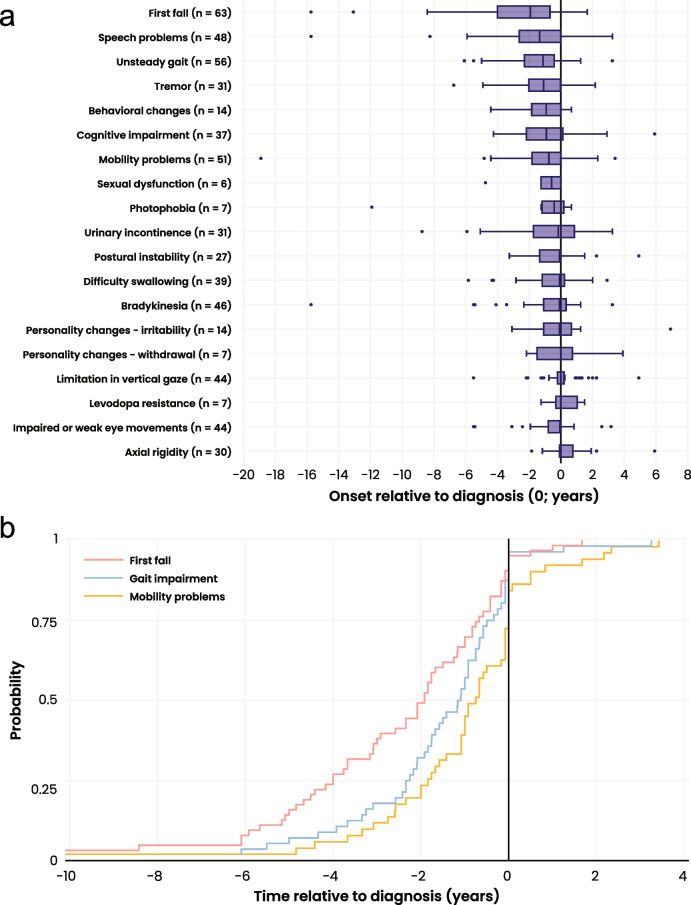
Onset of PSP symptoms relative to diagnosis. **a** Box plot showing the time of individual PSP symptom onset relative to diagnosis (0). The boxes only include participants whose date of symptom onset was known. Boxes are organized according to the median. The box plot shows 50% of the data within the box. The bottom of the box represents the 25th percentile (Q1), the top of the box represents the 75th percentile (Q3), and the solid line inside the box represents the median (Q2). The whiskers below the box indicate Q1–1.5*IQR, and the whiskers above the box represent Q3 + 1.5*IQR. Data points outside the box and whisker represent outliers. **b** Kaplan–Meier graph presenting the onset of key clinical milestones relative to the year of diagnosis, where year 0 indicates the year of diagnosis. Only participants with a documented date of symptom onset were included (falls: pink, *n* = 63, gait impairment: blue, *n* = 56, mobility problems: yellow, *n* = 51). Participants with symptom onset greater than 10 years prior to diagnosis (*n* = 3) are not shown for readability

Among symptoms with a documented date of onset in the medical records, all had a median onset that occurred prior to or at PSP diagnosis (Fig. 3A), with falling and speech problems/slurred speech/dysarthria occurring the earliest relative to diagnosis at medians of 2.0 (*n* = 63, IQR = 3.3 years; Fig. 3B, pink line) and 1.4 years prior (*n* = 48, IQR = 2.5 years), respectively. Among those who experienced unsteady gait or gait impairment and had a date of symptom onset documented in the medical record, the median onset of unsteady gait or gait impairment was 1.2 years before diagnosis (IQR: 1.8 years; Fig. 3B, blue line). Finally, among those who experienced mobility problems and had a date of symptom onset documented in the medical record, the median onset of mobility problems was 0.8 years before diagnosis (IQR: 1.8 years; Fig. 3B, yellow line).

**Fig. 4:**
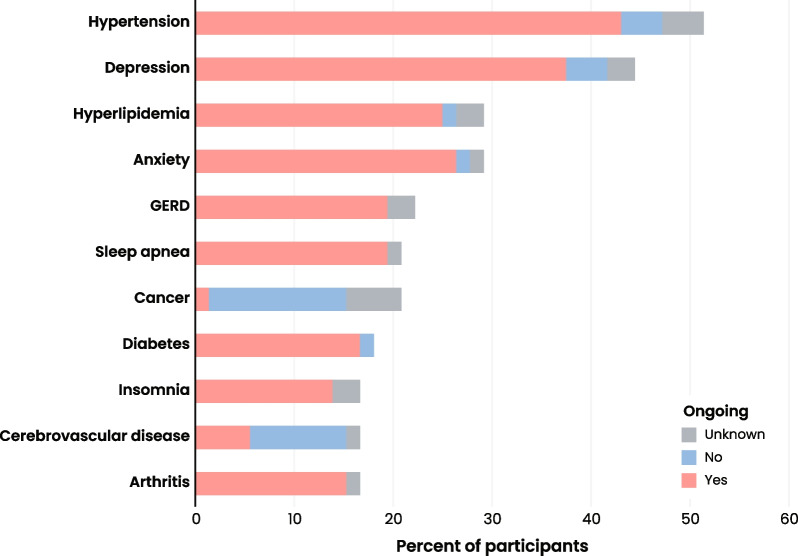
Status of participant comorbidities. Bar chart presenting the percent of participants with the most common comorbidities (*n* = 72). Comorbidities are shown as a function of whether they were ongoing as of the most recent relevant clinical record reviewed (ongoing = yes, pink; ongoing = no, blue; unknown status, gray). Only comorbidities documented for 10 or more participants are shown here. Abbreviations: gastroesophageal reflux disease, GERD. Cancer includes leukemia and lymphoma

Almost all participants (94.4%) had at least one documented comorbidity at any time pre- or postdiagnosis. Hypertension was the most common comorbidity (*n* = 37, 51.4%), followed by depression (*n* = 32, 44.4%) (Fig. 4, Additional file 3). Of the most common comorbidities, cancer (*n* = 15, 20.8%) was the only comorbidity that was not ongoing for the majority of affected participants (Fig. 4).

### Healthcare resource utilization (HRU)

**Fig. 5:**
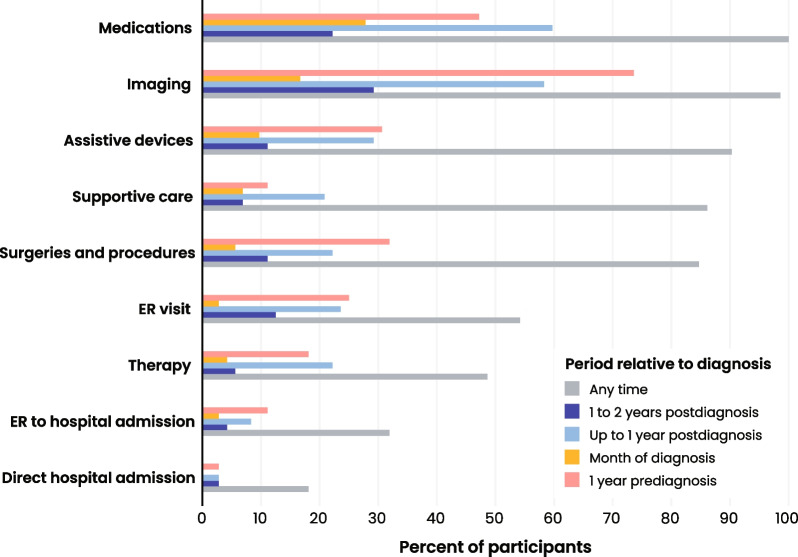
Cohort HRU (overall). Bar chart presenting participant use of healthcare resources at different timepoints prediagnosis and postdiagnosis (*n* = 72). Medications include all medications documented in the medical records. Resource utilization is shown at 1 year prediagnosis (pink), month of diagnosis (yellow), up to 1 year postdiagnosis (light blue), 1 to 2 years postdiagnosis (blue), and at any time in the medical records (gray). The use of a particular resource type is only documented in the time period when its use began. One year prediagnosis and up to 1 year postdiagnosis exclude data from the month of diagnosis

**Fig. 6:**
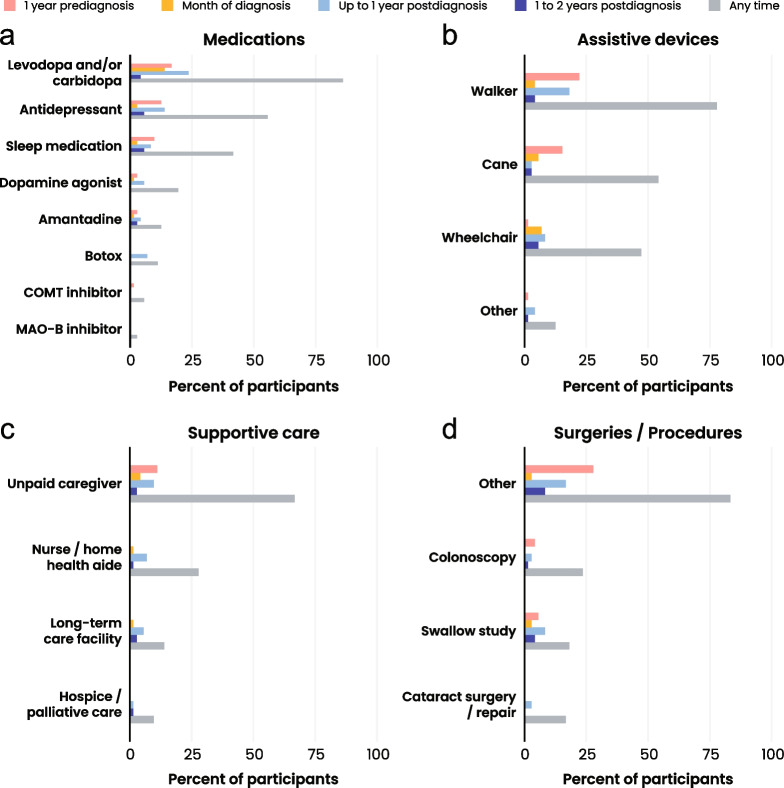
Cohort HRU. Bar charts presenting (**a**) most commonly prescribed PSP-related medications by date of prescription relative to diagnosis (*n* = 72). **b** Assistive devices by time started relative to diagnosis (*n* = 72). **c** Types of supportive care by time started relative to diagnosis (*n* = 72). **d** Top three most common types of surgeries and procedures performed at different timepoints relative to diagnosis (*n* = 72). Resource utilization is shown at 1 year prediagnosis (pink), month of diagnosis (yellow, up to 1 year postdiagnosis (light blue), 1 to 2 years postdiagnosis (blue), and at any time in the medical records (gray). The use of a particular resource type is only documented in the time period where its use began. One year prediagnosis and up to 1 year postdiagnosis exclude data from the month of diagnosis

Medications, imaging, assistive devices, supportive care, and surgeries and procedures were the most widely utilized healthcare resources, each of which was used by at least 85% of participants at some point during the disease course (Fig. 5, Additional file 4). All participants were prescribed at least one medication, and 47% of participants were prescribed a new medication in the year prior to diagnosis. The most commonly prescribed medications were levodopa with or without carbidopa (*n* = 62, 86.1%), antidepressants (*n* = 40, 55.6%), and sleep aids (*n* = 30, 41.7%) (Fig. 6A). Other common Parkinson’s medication classes were tabulated, but fewer than 20% of participants used these medications at any point. Ninety percent of participants had documentation of using at least one assistive device at any time during the disease journey (Fig. 5). Walkers were the most frequently used assistive device at any time during the disease journey (*n* = 56, 77.8%) and at 1 year prediagnosis (*n* = 16, 22.2%) (Fig. 6B). Canes and wheelchairs were also commonly used throughout the disease journey (*n* = 39, 54.2% and *n* = 34, 47.2%, respectively). Supportive care, defined as assistance received from a paid or unpaid caregiver in the home or in a residential or hospice facility, was documented in the medical records of 86.1% of participants (Fig. 5). The majority of participants received assistance from an unpaid caregiver in their home (*n* = 48, 66.7%), 27.8% (*n* = 20) received assistance from a nurse or home-health aide at home, 13.9% (*n* = 10) received care in a residential facility, and 9.7% (*n* = 7) received hospice care (Fig. 6C). Almost all assistance from an unpaid caregiver was provided by a family member (child, spouse or partner, sibling, in-law, or other family member). Most participants had at least one surgery or procedure (*n* = 61, 84.7%, Fig. 5), and the most common were colonoscopy (*n* = 17, 23.6%), swallow study (*n* = 13, 18.1%), and cataract surgery (*n* = 12, 16.7%) (Fig. 6D). The percentage of participants with at least one surgery or procedure decreased from 31.9% 1 year prediagnosis to 22.2% 1 year postdiagnosis. Inpatient visits were the least widely utilized healthcare resource, and utilization remained fairly constant over time; approximately one in five participants (*n* = 13, 18.1%) were directly admitted to the hospital, and one in three (*n* = 23, 31.9%) had emergency room (ER) visits that led to admission (Fig. 5, Additional file 4).

## Discussion

Here, we report a retrospective analysis of RWD to improve the understanding of PSP progression and management across healthcare settings and the disease journey. This study was unique in its tailored approach of partnering with PSP patient communities and individual advocates for participant recruitment. PSP-specific recruitment approaches, including hosting a live book reading and coauthoring blog posts with advocates for PSP, are detailed in the Methods section. Every step of the research process, from recruitment to account creation and research consent, was completed remotely through various modalities, including the AllStripes website, social media, internet advertising, email, and/or telephone. This approach reduced some of the logistical barriers to participating in research, as individuals with PSP could participate without traveling to research sites. This method, coupled with a direct partnership with PSP communities and advocates, enabled the recruitment of a larger cohort of PSP patients from a broader geographical area than many traditional site-based studies [1, 8, 9]. The short time for recruitment is another advantage of this approach compared to traditional site-based recruitment, which can take several years [1] and relies on patients attending appointments at participating research sites. Recruitment for in-person research studies is a particular challenge in studies of PSP due to the older age and potential mobility problems and difficulty traveling for PSP patients. Transportation is commonly cited as a barrier to participating in research for older individuals [22]. By consenting online to participate in medical records–based research, travel was removed as a barrier to participating in this study.

Medical records from multiple healthcare centers across the disease journey were collected, and data points were abstracted directly from these records, which allowed for broader data collection than many previous studies of PSP progression and management that relied on record collection from a single or limited number of care centers [4, 18–21]. Patients with rare diseases frequently have long and complex care journeys requiring medical appointments in multiple care settings, including community care providers, specialist centers, academic centers, and centers of excellence. Individuals may therefore have medical records located in a range of settings, and RWD studies focusing only on the medical records from larger medical centers omit a potentially significant portion of the individual’s care journey. The current study collected medical records from a mean of 2.9 healthcare centers per individual, with 23.6% of participants having documents in four or more healthcare centers, allowing for more comprehensive data capture of the participant’s medical history, management, and disease progression than would have been possible from a defined number of care centers.

Our findings are generally consistent with what has been previously reported in the literature. For this cohort, the median time from symptom onset to diagnosis was 3.7 years, which is consistent with other studies showing significant delays between symptom onset and PSP diagnosis of 3.5 to 4.9 years [4, 15, 19]. In agreement with previous reports of disease onset to symptom development [18], the earliest symptoms experienced by our cohort were falls and speech problems, where first fall was the most common symptom to occur at disease onset. The most common symptoms experienced prediagnosis and at any time during the disease course were very similar to other studies of PSP symptoms [4, 18, 19, 23], with falls, gait problems, and mobility problems as the most common symptoms prediagnosis and overall. Similar to other studies, speech problems and dysarthria were experienced by 79.2% of participants. However, 47.2% of all participants experienced these symptoms prior to diagnosis, in contrast with other studies, which report a smaller proportion of patients experiencing speech problems prior to diagnosis [19, 23]. This difference may arise from differences in milestone definition and/or date of symptomatic onset; for example, O’Sullivan et al. (2008) defined these milestones as “unintelligible speech” and “severe dysphagia” [19], which would likely have a later onset than an initial speech problem or dysphagia.

In addition to a high burden of symptoms associated with PSP, participants in our cohort also had documentation of many comorbidities, with hypertension and depression occurring in 51.4 and 44.4% of the cohort, respectively. While hypertension is a common comorbidity in the general population [24], a recent study demonstrated a modest yet significant association between hypertension and PSP and showed that the presence of hypertension at least 10 years prior to the onset of PSP symptoms was approximately 1.5 times more common in PSP cases than in matched controls [25]. Depression is also well recognized to be associated with PSP [26, 27]. Depression is more common in PSP patients than in the general population of adults 65 and over [28], affecting approximately 60% of PSP patients, as shown in a recent systematic review [27]. Hopelessness and withdrawal, specific subscales of depression, have also been shown to be higher in individuals with PSP than in those with other neurodegenerative diseases [29], and depression has been identified as an independent determinant of reduced quality of life in individuals with PSP [30]. The effects of a high comorbidity burden on increasing HRU are well documented for the general population [31, 32]; however, the specific impacts of multimorbidity on the HRU of patients with PSP have not yet been sufficiently investigated.

Very little research into the HRU of individuals with PSP has been published to date. A recent study analyzed claims data to quantify healthcare utilization among individuals with PSP and demonstrated significantly increased yearly healthcare spending for individuals with PSP compared to controls [33]. Our study adds to these findings by characterizing changes in HRU after individuals receive a diagnosis, including an increase in the utilization of assistive devices and supportive care that would be challenging to assess in claims or EHR databases. The majority of participants (86.1%) in our cohort were prescribed levodopa or carbidopa at any time. Given that up to half of individuals with PSP are initially misdiagnosed with Parkinson’s disease [15] due to the shared early clinical features of the two conditions, levodopa prescription before PSP diagnosis could be due to a misdiagnosis of Parkinson’s disease or part of a levodopa test to rule out Parkinson’s disease [34]. Approximately 30% of participants in this study still received levodopa after being diagnosed with PSP, despite one of the characteristics of PSP being poor levodopa responsiveness [35, 36]. It has been documented that some patients still derive modest benefit from levodopa or other dopamine agonists, particularly those with the PSP Parkinson phenotype [36–38]. Thus, some patients may continue on the drug postdiagnosis due to the potential for even a slight improvement, especially in the absence of a disease-modifying therapy. The PSP phenotype was not well documented in the records for most participants in the current study, suggesting that better education is required to make healthcare providers more aware of PSP phenotypes; however, it is likely that the majority of participants had the PSP-RS phenotype based on their reported symptoms and the fact that PSP-RS is the most common PSP phenotype [39].

PSP is well documented to result in functional disability. In a large cross-sectional study using physician-reported questionnaire data on over 800 patients with PSP across multiple countries, 60% of patients were using a nonwheelchair walking aid, and 23% were using a wheelchair at the time of analysis (median 31 months from symptom onset) [40]. In the current cohort, 90.3% of participants had documentation of using at least one assistive device during the course of their disease, with almost half requiring a wheelchair and nearly half using an assistive device prior to the month of PSP diagnosis. This degree of functional disability has a significant impact on the disease progression and prognosis for an individual with PSP. A previous study investigated the relationship between five clinical disability milestones, including the inability to walk unassisted, and reported a significant association between earlier occurrence of disability and shorter survival time, even after adjusting for sex and age at onset [20].

Due to the many motor, cognitive, neuropsychiatric, neurocognitive, and behavioral symptoms of PSP [41], it is unsurprising that many individuals with PSP require assistance from a caregiver. Most participants in this cohort required some form of supportive care (86.1%), most commonly assistance from an unpaid caregiver while living at home (66.7%). A recent study reported a similar proportion of PSP patients with a caregiver (73%), but only 24% of the cohort used a professional caregiver [39]. The majority of PSP patient caregivers in other studies were family members [41, 42], which is also consistent with our findings. This reality underscores the economic and psychosocial burden experienced by the families of those living with PSP.

While most participants in our cohort underwent at least one surgery or procedure (84.7%), two of the most common surgeries and procedures, cataract surgery and colonoscopy, are likely to be more age-related than PSP-specific [43, 44]. Alternatively, it is possible that cataract surgery may have resulted from a misdiagnosis of cataracts in PSP patients who had blurred or double vision related to their PSP, not cataracts. The second most common procedure documented was a swallow study, which is considered the gold standard diagnostic tool for the evaluation of dysphagia and is consistent with the high proportion of participants with dysphagia in this cohort. Dysphagia is a common symptom of PSP and increases the risk of aspiration, pneumonia, and death [45, 46]. As such, regular swallow studies are recommended to assess the risk of dysphagia and evaluate the patient’s need for diet modifications or feeding tube requirements [47].

RWD from medical records is characterized by several inherent limitations that may have impacted the results of this study. While every effort was made to collect the complete medical record of every participant in the study, some documents may have been lost, destroyed, or were otherwise irretrievable from participants’ healthcare centers. Data quality depended primarily on the accurate reporting of information on clinical symptoms by neurologists and movement disorder specialists, which may be prone to clinician error, differences in documentation processes, or incomplete reporting. The postdiagnosis follow-up time was not equal and varied across all participants from 0 months to over 5 years, which may have impacted our findings since individuals were likely at different stages of their disease progression during the study period. In addition, this study may be characterized by potential selection bias since recruitment of participants relied heavily on partnership with PSP patient communities and individual advocates. As such, recruitment was biased toward English-speaking individuals who were connected with the wider PSP patient community with access to a computer and the internet. Finally, only individuals who had received a diagnosis of PSP and had not participated in an interventional trial for PSP were eligible for inclusion in the study. Therefore, undiagnosed and misdiagnosed individuals and those who previously participated in an interventional trial were not captured in this analysis.

## Conclusion

This novel retrospective study adds to our current knowledge and understanding of PSP symptoms, comorbidities, and HRU across the disease journey. While previous studies have reported similar findings, this study was unique in the richness of clinical data collected. Data were abstracted from medical records collected from multiple patient healthcare centers, allowing for more comprehensive data capture across the spectrum of healthcare settings in which individuals with PSP receive care. Additionally, the distinctive approach to patient recruitment yielded a fairly large cohort for a rare disease, enabling individuals to participate in research without the need for travel. Individuals with PSP and their legally authorized representatives also gave consent to be recontacted, allowing for ongoing engagement with this cohort for future PSP research opportunities. This approach to patient recruitment, engagement, and collection of RWD is applicable to a multitude of rare diseases beyond PSP and may help to advance the understanding of many aspects of rare diseases, from initial symptoms and presentation to management and HRU under current standards of care.

## Methods

AllStripes is a medical data company specializing in rare diseases [48]. Patients and legally authorized representatives who join an AllStripes research program can access their digitized and organized medical records via a secure platform through which they can provide consent for data in their records to be used in research studies. Individuals with PSP from the US, UK, and Canada were recruited to join the AllStripes platform via digital marketing between 27 March 2019 and 31 December 2021. A tailored approach was implemented to recruit individuals with PSP by working closely with AllStripes Ambassadors for PSP (patients and caregivers who choose to participate in individual advocacy and share their experiences with PSP) and patient advocacy groups (CurePSP and the PSP & CBD Foundation). Recruitment tools developed specifically to engage individuals with PSP included search engine marketing, sharing AllStripes Ambassador stories on the AllStripes website [49–51], coauthoring a blog post with two PSP advocates [52], cohosting the live reading of a book authored by an AllStripes Ambassador for PSP [53], and interviewing an AllStripes Ambassador about their experience with the AllStripes research program for PSP [54]. Each of these recruitment tools was shared on social media and with patient advocacy groups, and AllStripes Ambassadors for PSP were able to share these tools within private community groups for PSP if they chose to do so.

Patients or their legally authorized representatives were asked to sign a Health Insurance Portability and Accountability Act of 1996 (HIPAA) release form (US and Canada) or Subject Access Request (SAR) form (UK), provide a list of hospitals and clinics where the individual received care, and sign a research consent form (to allow for deidentified data from medical records to be used in minimal risk research and for participants to be recontacted) approved under a central institutional review board (IRB). Participants were also able to add a care partner to their account — a trusted person to help manage or eventually take over their account if the individual with PSP was no longer able to manage their account independently. Participant medical records were requested from all healthcare centers reported by the participant or legally authorized representative, including academic centers of excellence and community healthcare settings. Medical records were digitized, organized, and made available to participants on the secure AllStripes platform. Predefined key data elements were captured from the medical records by trained clinical abstractors in a structured format. During data abstraction, an electronic audit trail of each data point to the source documents was maintained for quality assurance purposes. Abstracted, deidentified data were evaluated by clinical abstractors and researchers to ensure quality, and the final data were exported for analysis.

Participants from the registry of individuals with PSP who consented to research on the AllStripes platform were included in this study if they had at least one neurology record within the past year and if clinical notes in their medical record had documentation of a confirmed, probable, likely, suggestive, or possible PSP diagnosis given by a neurologist or movement disorder specialist. Individuals who reported participation in an interventional clinical trial for the treatment of PSP at or before enrollment on the AllStripes platform or had documentation of PSP trial participation in their medical records were excluded from the analysis. Previous participation in a clinical trial that was not for the treatment of PSP did not exclude individuals from the study. While individuals from the UK could join the AllStripes program, only participants from the US and Canada were eligible for inclusion in this study. Any data elements that were not documented in the participant’s medical record were reported as missing data, and only participants who had a documented date of symptom onset were included in the respective Kaplan–Meier curves.

Demographic variables were collected via a demographics survey when the participant or legally authorized representative joined the AllStripes research program and included gender, age, and country of residence at sign-up. These demographic variables were further verified via comparison with data recorded in the participant’s medical records if data captured in the survey were unclear or missing. Abstracted clinical variables included PSP phenotype, diagnosis date, age at diagnosis, and diagnosing physician specialty. Disease onset was defined as the first documentation of clinical symptom presentation, and the diagnosis date was based on verification of PSP diagnosis in the clinical notes from a neurologist or movement disorder specialist. A targeted list of PSP symptoms was developed based on subject matter expertise and a review of the literature (Additional file 1), and comorbidities were defined as any recorded diagnosis that fell outside this list of PSP symptoms. Falls and gait abnormalities were captured separately. Symptoms and comorbidities were reported as the number and percent (n, %) of participants with the symptom or comorbidity of interest. Symptoms and comorbidities were classified into the following categories according to their date of onset: before diagnosis (prediagnosis), month of diagnosis, after diagnosis (postdiagnosis), and any time during the disease journey (any time). Comorbidities were characterized as ongoing for the data analysis if the condition was referenced as of the most recent relevant clinical note reviewed. All available medical history was included for abstracting data from the medical records and populating these variables. Incomplete dates were imputed to the first day of the month if the day was unknown or first day of the seventh month if the month was unknown. Study years were calculated by dividing the total number of days by 365.25.

Key clinical outcomes included hospitalizations, surgeries and procedures, imaging, assistive devices, medications, therapies, and supportive care. Supportive care was defined as assistance received from a paid or unpaid caregiver in the home or in a residential or hospice facility. Outcomes were reported as the number and percent (n, %) of participants with the outcome of interest. All outcomes were reported at the following time points: 1 year prediagnosis, month of diagnosis, up to 1 year postdiagnosis, and 1 year postdiagnosis to 2 years postdiagnosis. Each time-to-event variable was measured as the number of days between the date of PSP diagnosis and the date the symptom was first documented in each participant’s records. Participants were censored at death (*n* = 3), when lost to follow-up, or at the end of the study period (between May 2020 and April 2022, depending on the date of last review for each patient). Data from all participants meeting the inclusion criteria were analyzed using R (version 4.2.1). Only descriptive methods were employed in this study, and hence, no formal association testing was performed.

### Supplementary Information

**Additional file 1.** Targeted list of PSP symptoms

**Additional file 2 **Number and percent of participants with given symptom presentation, prediagnosis, month of diagnosis, postdiagnosis, or at any time, as documented in the medical records (*n*=72)

**Additional file 3 **Number of participants with documentation of comorbidities at any time pre- or postdiagnosis (*n*=72). Only comorbidities affecting three or more patients are shown

**Additional file 4.** Participant use of healthcare resources at different timepoints prediagnosis and postdiagnosis (*n*=72). One year prediagnosis and up to 1 year postdiagnosis exclude data from the month of diagnosis

## Data Availability

The datasets generated during the current study are not publicly available due to the data containing information that could compromise research participant privacy. However, they may be available through an application process managed by AllStripes Research and limited to researchers working with recognized academic or research organizations who agree to protect the privacy of participants. To decrease the risk of participant reidentification, certain information may be redacted from any data shared, and dates may be shifted and/or provided with decreased granularity. Requests for data access may be directed to research@allstripes.com.

## Abbreviations

EHR: Electronic health record
ER: Emergency room
HIPAA: Health Insurance Portability and Accountability Act of 1996
HRU: Healthcare resource utilization
IRB: Institutional review board
PSP: Progressive supranuclear palsy
PSP-P: PSP-predominant parkinsonism
PSP-RS: PSP-Richardson syndrome
RWD: Real-world data
SAR: Subject access request
